# Diagnostic value of neutrophil-to-lymphocyte, lymphocyte-to-monocyte and platelet-to-lymphocyte ratio among patients with COVID-19 pneumonia: A retrospective study

**DOI:** 10.12669/pjms.38.5.5798

**Published:** 2022

**Authors:** Xiaoping Long, Ting Zhang, Shan Duan

**Affiliations:** 1 Xiaoping Long, Department of Pulmonary and Critical Care Medicine, The First Affiliated Hospital of University of South China, Hengyang 421001, Hunan Province, P.R. China; 2 Ting Zhang Department of Pulmonary and Critical Care Medicine, Affiliated Nanhua Hospital of University of South China. Hengyang 421002, Hunan Province, P.R. China; 3 Shan Duan, Department of Pulmonary and Critical Care Medicine, The First Affiliated Hospital of University of South China, Hengyang 421001, Hunan Province, P.R. China

**Keywords:** COVID-19, Lymphocyte count, Neutrophil-to-lymphocyte ratio, Lymphocyte-to-monocyte ratio, Platelet-to-lymphocyte ratio

## Abstract

**Objectives:**

Our study was aimed to investigate the clinical characteristics of the patients with COVID-19 pneumonia and research new diagnostic methods for the disease.

**Methods:**

In this retrospective study, medical records of 46 novel coronavirus-infected pneumonia (NCIP) patients and 30 healthy individuals in the two multiple hospitals from January 2020 to March 2020 were studied. Clinical characteristics, chest computed tomographic (CT) scans, medicine treatment and laboratory information were collected and retrospectively analyzed. Neutrophil-to-lymphocyte ratio (NLR), lymphocyte-to-monocyte ratio (LMR) and platelet-to-lymphocyte ratio (PLR) were evaluated.

**Results:**

The main symptoms of the patients with NCIP were fever (100%), cough (82.6%), anorexia (37%), expectoration (34.8%) and fatigue (21.7%), dyspnea (15.2%). Ground glass opacity (GGO) with patch shadow was the main observation of the CT imaging (43.4%), followed by GGO (21.7%), patch shadow (19.5%), GGO with consolidation (8.7%) and GGO with reticular pattern (2.1%). The median white blood cell (WBC) count, lymphocyte count, platelet, and lymphocyte-monocyte ratio (LMR) in NCIP group were all significantly lower than in control group (p<0.001, for all comparisons), while the median neutrophil-monocyte ratio (NLR) and platelets-monocyte ratio (PLR) were both significantly higher (p<0.001, for both comparisons). Median WBC count, lymphocyte count, and platelet count on discharge were significantly higher than on admission (p<0.05). Median PLR was significantly lower two weeks after discharge (p<0.001), while NLR remained the same. The area under the curve (AUC) value of WBC, lymphocyte and platelet counts, NLR, LMR and PLR were 0.766, 0.931, 0.655, 0.780, 0.847 and 0.845, respectively. Early stages of the disease were associated with quick changes in WBC, lymphocyte, and platelet levels. However, NLR did not recover even two weeks after the discharge.

**Conclusion:**

Changes in WBC, lymphocyte, and platelet counts, as well as NLR, LMR and PLR are strongly associated with COVID-19 pneumonia. Monitoring blood markers may assist in evaluating the progression of the disease

## INTRODUCTION

The outbreak of Coronavirus Disease 2019 (COVID-19), causing novel coronavirus-infected pneumonia (NCIP), started in China in December 2019. Although comprehensive epidemic prevention measures and establishment of a leading group of experts for epidemic response in China were able to successfully control the outbreak, it quickly turned into a global pandemic. As of November 1, 2021, there had been 246,713,220 confirmed cases of COVID-19, including 5,000,000 deaths, reported to WHO. COVID-19 caused serious acute lung injury and multi-organ failure, with many patients dying quickly of acute respiratory failure. Therefore, it became crucial to identify early markers for diagnosing and predicting the severity of the disease.

Previous studies had shown that COVID-19-infected adult exhibited reduced lymphocyte and elevated platelet counts although there was no decrement in children.^1^-^3^ These observations suggested that platelet-to-lymphocyte ratio (PLR) might be a new indicator in the monitoring of patients with COVID-19.^2^,^4^,^5^ Clinical research of 456 patients with COVID-19 showed that severe cases had higher neutrophil-to-lymphocyte ratio (NLR) and lower lymphocyte count than less severe cases.^6^ Furthermore, it had a statement that NLR was significantly associated with disease severity and mortality.^7^ The Previous studies reported that lower lymphocyte-to-monocyte ratio (LMR) correlated with the disease severity in the cases of respiratory virus infections and could be used to discriminate between the pneumococcal pneumonia and influenza.^8^-^10^ Therefore, it is plausible that those blood markers might also be associated with COVID-19 infection.

In our study, we retrospectively evaluated levels of NLR, LMR and PLR in patients with COVID-19. Our aim was to investigate the variation trend of those blood tests results in patients and healthy volunteers and evaluate the changes before and after the treatment.

## METHODS

In this retrospective study, medical records of 46 patients with confirmed COVID-19 pneumonia, admitted in the First Affiliated Hospital, University of South China and Affiliated Hospital Nanhua, University of South China (Hunan, China) from January 2020 to March 2020, were retrospectively reviewed. Medical records of 30 healthy individuals were obtained from the Affiliated Hospital Nanhua, University of South China in the same time period. The Medical Ethics Committee of the First Affiliated Hospital of University of South China approve this study (no. 20201201001, date: 2020 March 20^th^). Based on the diagnosis, patients were divided into two groups, NCIP group and control group. The composite endpoint of the study was March 19, 2020. The results of RT-PCR assay in all cases were negative after discharge. Two patients in the study had repeated positive RT-PCR on 2-week follow-up. The results of the routine blood tests of these patients were not included.

According to the COVID-19 diagnosis and treatment scheme issued by the seventh Revised Trial Version of the Novel Coronavirus Pneumonia Diagnosis and Treatment Guidance,^11^ patients’ conditions were classified as common (mild), severe and critical.

Clinical and laboratory information, and CT scans were retrieved from the hospital electronic database. All cases had CT scans and RT-PCR tests performed before admission and two weeks after the discharge from the hospital. Clinical information included demographic data, medical history, symptoms, signs, course of the disease, length of hospital stay, therapeutic schedule and basic disease. Blood tests upon admittance and in the early-stage evaluation (24-48 hours) were carried out, including WBC count, neutrophil count, lymphocyte count, monocyte count, platelet count and PaO_2_. According to the tests, NLR, LMR, PLR and PaO_2_ /FiO_2_ ratio were measured.

Categorical variables were presented as frequency rates and percentages, and continuous variables- as mean and standard deviation (SD), median and interquartile range (IQR) values. The independent sample t test and nonparametric two-tailed Student t test was used to analyze variables. The dualistic logistic regression analysis and the receiver operator characteristic curve (ROC) were used to get the optimal cut-off value, sensitivity (sensitivity). *P*<0.05 was considered statistically significant.

## RESULTS

Among patients in the NCIP group, 39 cases (84.8%) were classified as a common (mild) group and seven cases (15.2%) as the severe group. Forty-four cases (95.7%) had exposure history. In our study, 38 patients (82.6%) were younger than 65 years and 31 patients (67.6%) were males. Twenty-two patients (47.8%) had at least one underlying disease, such as diabetes (15.2%), hypertension (10.8%) and coronary heart disease (10.8%). The main symptoms were fever (100%), cough (82.6%), anorexia (37%), expectoration (34.8%) and fatigue (21.7%). Only seven patients felt dyspnea. The temperature of 33 patients (82.6%) were lower than 38.5°C. We estimated the median time from symptom onset to diagnosis was 5 days (IQR 3-9 days) and the average hospital stay of patients was 13 days (Table-I).

**Table I:** Demographics and clinical characteristics in 46 NCIP patients.

Variables	n	n%
**Age (years)**		
>65	8	17.4
≤65	38	82.6
**Gender**		
Male	31	67.6
Female **(comorbidities)**	15	32.4
Hypertension	5	10.8
Diabetes	7	15.2
Coronary heart disease	5	10.8
Chronic lung disease	1	2.1
Chronic renal disease	0	0
Cancers*	0	0
Chronic liver disease	3	6.5
Cerebrovascular disease	1	2.1
**Initial symptoms**		
Fever	46	100
≥38.5	13	28.3
<38.5	33	71.7
Cough	38	82.6
Expectoration	16	34.8
Pharyngodynia	4	8.7
Headache or dizzy	5	10.8
Fatigue	10	21.7
Dyspnea	7	15.2
Nausea or vomiting	6	13
Diarrhea	1	2.1
Anorexia	17	37
**Clinical classification**		
Common (Mild) group	39	84.8
Severe group	7	15.2
Critical group	0	0
Exposure history	44	95.7

***Note:***

* Cancers included leukemia and lymphoma

All patients received high resolution CT (HRCT) of the chest within 24 hours after admission. Most patients (71.7%) had double lung lesions. Right lower lobe or left lower lobe lesions were found in only three patients (6.5%, each). Lung lesions of five patients (10.9%) distributed in subpleural area and three patients (6.5%) in peripheral. Ground glass opacity (GGO) with patch shadow was the main CT imaging finding (43.4%). GGO in 10 patients (21.7%), patch shadow in nine patients (19.5%), GGO with consolidation in 4 patients (8.7%) and GGO with reticular pattern in one patient (2.1%) were diagnosed. The improvement ratio of CT imaging changes 6 days after the admission was only 58.6% (Table-II).

**Table II:** Characteristics of CT scans in 46 NCIP patients.

Variables	n	n%
**Involved lung zones**		
Right upper lobe	1	2.1
Right middle lobe	0	0
Right lower lobe	3	6.5
Left upper lobe	0	0
Left lower lobe	3	6.5
Double lung	33	71.7
**Predominant distribution**		
Subpleural area	5	10.9
Peripheral	3	6.5
Central	0	0
**Radiological features**		
GGO	10	21.7
GGO+ Consolidation	4	8.7
Consolidation	0	0
Patch shadow+ GGO	20	43.4
Patch shadow	9	19.5
Reticular pattern+ GGO	1	2.1

***Abbreviations:*** GGO, ground glass opacity.

Antiviral treatment of the NCIP was implemented in majority of the cases. 44 patients (95.7%) received lopinavir and ritonavir, and 39 patients (84.8%) received recombinant human interferon α2b. All patients received more than two antiviral drugs. Fourteen patients (30.4%) received immunoglobulin with antiviral drugs. five patients (10.9%) received corticosteroid with antiviral drugs, and 37 patients (80.4%) received traditional Chinese medicine with antiviral drugs.

**Fig.1:**
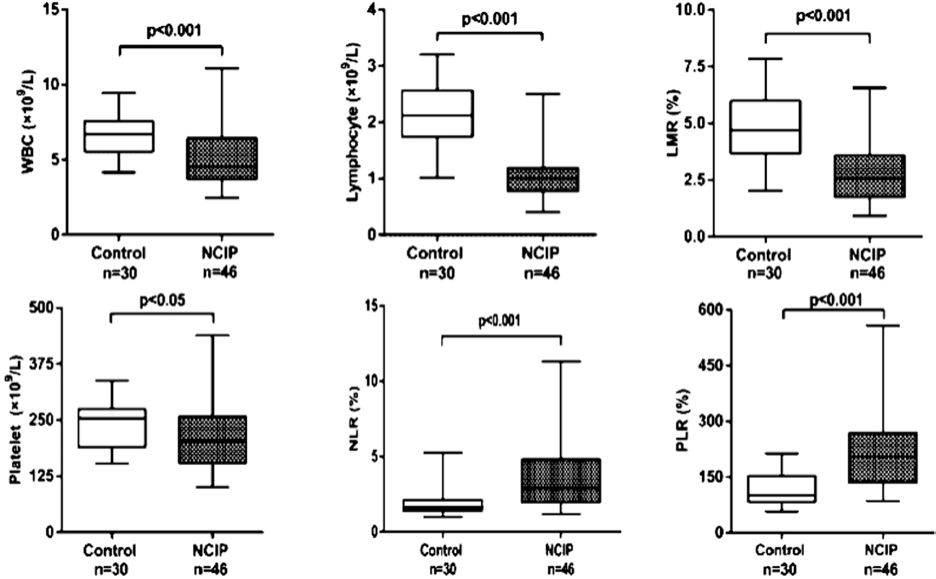
Comparison of blood markers in 30 healthy controls and in 46 NCIP patients.

The routine blood tests were recorded 24-48 hours after the admission. There was no difference in the percentage of males, age and BMI between NCIP and control groups (p>0.05). Median WBC count (4.53×10^9^/L) (Fig.1A.), lymphocyte count (1.01×10^9^/L) (Fig.1B.), platelet count (202.50×10^9^/L) (Fig.1C.), and LMR (2.58%) (Fig.1E.) were significantly lower in the NCIP group (p<0.001), while the median NLR (2.89%) (Fig.1D.) and PLR (202.67%) (Fig.1F.) were significantly higher (p<0.001) (Table-III).

**Table III:** Demographic and laboratory measurements in NCIP patients and volunteers (admission to hospital in 24-48hours).

Variables	NCIP group n=46	Control group n=30	p value
Age (years)	44.63±15.55	46.37±17.59	0.653
Male (n, n%)	31(67.4%)	16(53.3%)	0.223
BMI (kg/m^2^)	22.08±3.99	23.62±3.19	0.081
WBC (×10^9^/L)	4.53(3.70-6.46)	6.70(5.55-7.56)	<0.001
Neutrophil (×10^9^/L)	2.99(2.10-4.73)	3.53(2.99-4.67)	0.121
Lymphocyte (×10^9^/L)	1.01(0.78-1.19)	2.12(1.75-2.57)	<0.001
Monocyte (×10^9^/L)	0.40(0.30-0.50)	0.40(0.40-0.50)	0.191
Platelet (×10^9^/L)	202.50(154.25-258.00)	254.00(189.75-275.25)	0.023
NLR(%)	2.89(1.97-4.83)	1.62(1.40-2.13)	p<0.001
LMR(%)	2.58(1.74-3.59)	4.70(3.68-6.00)	p<0.001
PLR(%)	202.67(134.90-264.52)	101.13(83.18-153.33)	p<0.001

Unless otherwise indicated, values are expressed as mean ± SEM, median and IQR.

**Abbreviations:** BMI, body index; WBC, white blood cell; NLR, neutrophil-to-lymphocyte ratio;

**Fig.2:**
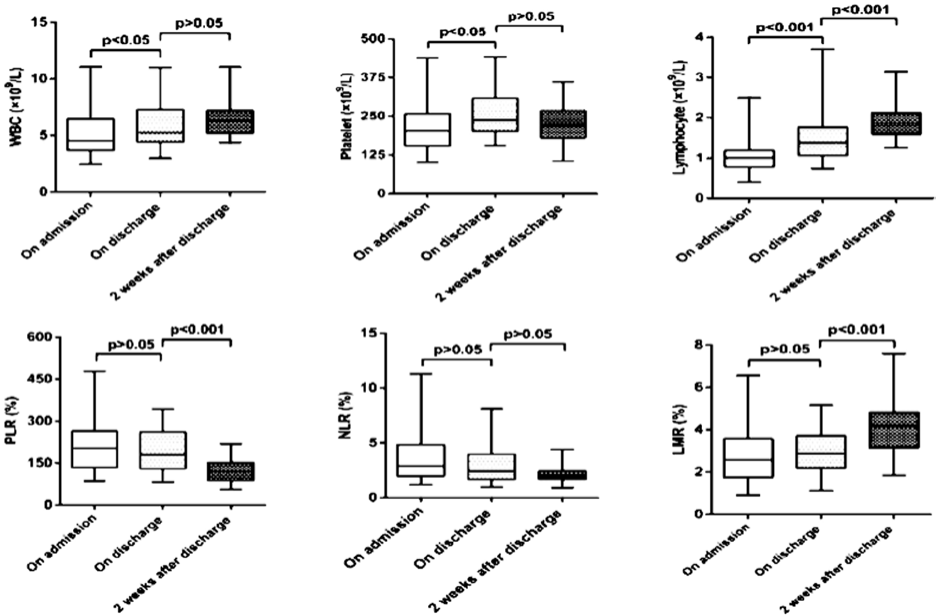
Comparison of blood markers in three time periods.

LMR, lymphocyte-to-monocyte ratio; PLR, platelet-to-lymphocyte ratio;

Median WBC count (5.26×10^9^/L) (Fig.2A.), lymphocyte count (1.38×10^9^/L) (Fig.2B.) and platelet count (237.00×10^9^/L) (Fig.2C.) on discharge were significantly higher than on admission (p<0.05). The median lymphocyte count (1.86×10^9^/L) and LMR (4.18%) (Fig.2E.) two weeks after discharge were significantly higher than on discharge (p<0.001), while median PLR (118.80%) (Fig.2F.) was significantly lower two weeks after discharge (p<0.001). There was no significant difference in neutrophil count and NLR (Fig.2D.) in all three time periods (p>0.05) (Table-IV)

**Table IV:** Laboratory measurements of 44 NCIP patients in three time periods.

Variables	On admission	On discharge	two weeks after discharge
WBC (×10^9^/L)	4.53(3.70-6.46)*	5.26(4.45-7.29)	6.26(5.21-7.20)
Lymphocyte (×10^9^/L)	1.01(0.78-1.19)**	1.38(1.07-1.77)^##^	1.86(1.59-2.13)
Platelet (×10^9^/L)	202.50(154.25-258.00)*	237.00(202.00-308.50)^#^	222.00(180.00-267.50)
NLR(%)	2.89(1.97-4.83)	2.42(1.67-3.95)^##^	2.05(1.68-2.43)
LMR(%)	2.58(1.74-3.59)	2.87(2.19-3.73)^##^	4.18(3.14-4.82)
PLR(%)	202.67(134.90-264.52)	180.00(129.62-260.84)^##^	118.80(88.46-152.39)

Unless otherwise indicated, values are expressed as mean ± SEM, median and IQR.

* :p<0.05,

** :p<0.001, On admission vs On discharge.

# :p<0.05,

## :p<0.001, On discharge vs two weeks after discharge.

***Abbreviations:*** WBC, white blood cell; NLR, neutrophil-to-lymphocyte ratio;

LMR, lymphocyte-to-monocyte ratio; PLR, platelet-to-lymphocyte ratio.

**Fig.3:**
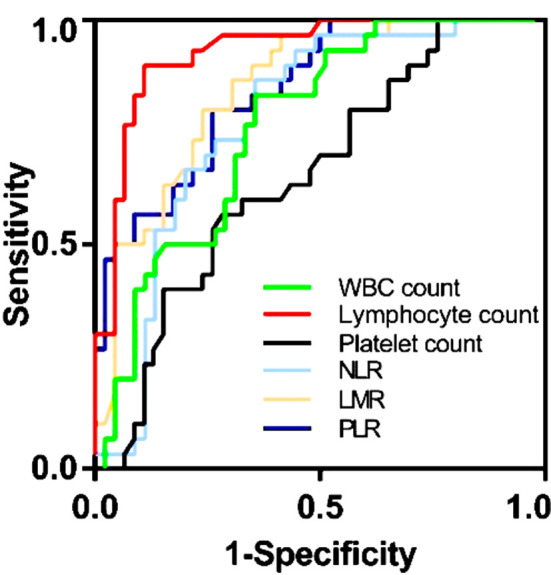
The ROC curves of WBCs, lymphocytes, platelets, NLR, LMR and PLR.

We performed dualistic logistic regression analysis and calculated the receiver operator characteristic curve (ROC) for relationship between blood markers and diagnosis. ROC analysis at a cut-off level of < 5.175×10^9^/L WBC count showed the best sensitivity (64.4%) and specificity (83.3%) to make a best diagnosis (AUC=0.766). Similar results were seen for lymphocyte count (cut-off < 1.425×10^9^/L, sensitivity 89.1%, specificity 90%, AUC=0.931), platelet count (cut-off < 251.50×10^9^/L, sensitivity 71.7%, specificity 56.7%, AUC=0.655), NLR (cut-off >2.306%, sensitivity 64.4%, specificity 86.7%, AUC=0.780), LMR (cut-off < 3.24%, sensitivity 69.6%, specificity 86.7%, AUC=0.847) and PLR (cut-off >156.189%, sensitivity 73.9%, specificity 80%, AUC=0.845) (Table-V, Fig.3.).

**Table V:** Sensitivity and specificity of blood markers for the diagnosis.

Variables	AUC	Cut-off value	Sensitivity (%)	Specificity (%)
WBC (×10^9^/L)	0.766	5.175	64.4	83.3
Lymphocyte (×10^9^/L)	0.931	1.425	89.1	90.0
Platelet (×10^9^/L)	0.655	251.50	71.7	56.7
NLR (%)	0.780	2.306	64.4	86.7
LMR (%)	0.847	3.24	69.6	86.7
PLR (%)	0.845	156.189	73.9	80.0

***Abbreviations:*** WBC, white blood cell; NLR, neutrophil-to-lymphocyte ratio;

LMR, lymphocyte-to-monocyte ratio; PLR, platelet-to-lymphocyte ratio;

## DISCUSSION

Most of the cases in the NCIP group in the current Hengyang study were not serious, and presented with the same symptoms (fever, cough, and fatigue) as patients in Wuhan.^12^ However, these symptoms are also characteristic for common pneumonia or influenza, which makes diagnosis of COVID-19 by symptoms alone difficult. Our study showed that, similarly to the reports from Wuhan,^13^ median time from symptom onset to diagnosis was five days (IQR 3-9 days) and the average hospital stay of patients was 13 days. We showed that 35 patients (75%) were not hospitalized until day-9 of the disease, and the patients were hospitalized for over seven days. These results provide further evidence of the difficulty in identifying and giving timely treatment at an early stage of COVID-19 disease and emphasize the importance of developing new diagnostic tools.

Blood tests and chest CT are used as the first step of COVID-19 diagnostics. If the patients have the exposure history or typical CT images, the RT-PCR for nasal and pharyngeal swab specimens are used as the second step, especially when there is a shortage of RT-PCR kits. Therefore, it is very important to obtain maximum information from the blood tests and chest CT. We found that CT images of patients with NCIP were different from the patients with common pneumonia and influenza. In our study, most of the patients had double lung lesion. Pure ground-glass opacity (GGO) with patch shadow (43.4%) was the hallmark of the NCIP in Hengyang. Some reports showed that GGO was found at the early stage of the disease and is considered the earliest CT manifestation of COVID-19.^14^ Studies also show that consolidations (including multifocal, patchy or segmental consolidation) are usually present in NCIP patients, and may be associated with the progression of the disease.^15^,^16^ There were only 4 patients with GGO and consolidation in our study, which suggest that most Hengyang patients were in the very early stages of the disease. Importantly, the radiological finding like GGO and consolidation are also found on CT images of H1N1-infected patients.^17^ The clinical symptoms, including fever, cough, and dyspnea, are also characteristic of HINI infection.^14^ Therefore, it is important to be able to distinguish between the H1N1 and NCIP diagnoses.

Although there is no effective treatment for the COVID-19 infection, we wanted to analyze treatment options, available in Hengyang hospital for accumulated experience. Some reports suggested that antiviral agentslopinavir/ritonavir had a potential affinity for COVID-19 helicase.^16^ In our study, antiviral treatment of NCIP in included ribavirin, oseltamivir, recombinant Human Interferon α2b, arbidol and lopinavir/ritonavir. More than 80% of the patients were treated with recombinant Human Interferon α2b and lopinavir/ritonavir with no reported side effects. In addition, 80.4% patients were treated with traditional Chinese medicine. Similarly, there were no reports of side effects.^18^ All the patients were discharged with no symptoms and negative RT-PCR tests, indicating that the received NCIP treatment was effective and reliable.

Decreased levels of lymphocytes and neutrophils are found in COVID-19-infected patients.^19^ Some reports showed that lymphocyte level at admission was related to the prognosis, while elevated platelets and higher PLR were associated with the longer average hospitalization time.^2^ It had been shown that in COVID-19 patients higher NLR was significantly associated with an increased risk of all-cause death during hospitalization.^20^,^21^ It also showed that NLR had an significant correlation with disease severity.^7^ WBC, NLR, LMR and PLR of patients with severe disease were significantly higher than those of non-severe patients.^22^ In our study, we compared the levels of blood markers in NCIP patients and healthy individuals, and evaluated the changes in these parameters at three time points (admission, discharge and two weeks follow-up). We found that WBC, lymphocyte, and platelet counts, NLR, LMR and PLR in NCIP patients differed significantly from those of healthy individuals. While WBC count, lymphocyte count, LMR and platelet count significantly decreased in NCIP group, NLR and PLR were significantly elevated. We may speculate, therefore, that the levels of these blood cells and markers might be associated with the development of the COVID-19 infection.

Our results indicate that the median WBC and lymphocyte counts of NCIP patients gradually increased from discharge to two weeks after discharge. Platelet count elevated quickly on discharge but remained unchanged two weeks after discharge. There was no significant difference in LMR and PLR until two weeks after discharge, while NLR remained unchanged through all three time periods.

We used dualistic logistic regression analysis and ROC curves to find the factors, related to the diagnosis of NCIP. WBC count, lymphocyte count, platelet count, NLR, LMR and PLR in our study were closely associated with the diagnosis of the NICP, with AUC values of over 0.7. The sensitivity of lymphocyte count was 89.1% and the specificity was 90%. Our results suggest that these blood markers might help for diagnosing and observing the progression of COVID-19 infection.

### Limitations:

This is a retrospective study in the small city of China, with a small sample size. During the same period, there were no critical patient and all patients had great outcome. Therefore, we only analyzed the data from non-critical patients. Larger-scale studies and clinical trials, with patients with different disease severity are needed to further assess the diagnostic and prognostic value of different blood markers in COVID-19 NCIP patients.

## CONCLUSIONS

Our study showed that WBC, lymphocyte, and platelet counts, as well as NLR, LMR and PLR are closely associated with the disease in NCIP patients. Early stages of the disease are associated with quick changes in WBC, lymphocyte, and platelet levels. However, NLR does not recover two weeks after the discharge. Therefore, monitoring blood markers, such as WBC, lymphocytes and platelets may assist in evaluating the progression of the disease. Our findings may provide new convenient method to discover and diagnose the disease especially in countries with limited medical resources.

### Abbreviations:

**COVID-19:** Coronavirus disease in 2019,

**NCIP:** Novel coronavirus infected pneumonia,

**HRCT:** High resolution computed tomographic,

**WBC:** White blood cell,

**NLR:** Neutrophil-to-lymphocyte ratio, LMR: Lymphocyte-to-monocyte ratio,

**PLR:** Platelet-to-lymphocyte ratio,

**GGO:** Ground glass opacity,

**PaO_2_:** Partial pressure of arterial oxygen,

**IQR:** Interquartile range,

**RT-PCR:** Real-time reverse-transcriptase polymerase-chain reaction, **FiO_2_:** Oxygen concentration,

**SD:** Standard deviation,

**CDC:** Chinese Center for Disease Prevention and Control, **ROC:** Receiver operator characteristic curve.

### Authors’ contribution:

**XL & TZ:** Conceived and designed the study.

**TZ & SD:** Collected the data and performed the analysis.

**SD:** Was involved in the writing of the manuscript and is responsible for the integrity of the study.

All authors have read and approved the final manuscript.
